# Reduced coronary flow reserve in Anderson-Fabry disease measured by transthoracic Doppler echocardiography

**DOI:** 10.1186/1476-7120-3-11

**Published:** 2005-04-27

**Authors:** Paweł Petkow Dimitrow, Marek Krzanowski, Anetta Undas

**Affiliations:** 1 2nd Department of Cardiology, Collegium Medicum Jagiellonian University, Cracow, Poland; 2 2nd Department of Internal Medicine, Collegium Medicum Jagiellonian University, Cracow, Poland

## Abstract

Coronary flow reserve was assessed in a patient with Anderson-Fabry disease complicated by symmetric left ventricular hypertrophy. Coronary flow reserve was measurable in all three major coronary arteries providing an opportunity to compare regional coronary flow reserve from different vascular beds. In this patient all the three vascular beds supplied diffusely hypertrophied myocardium. Coronary flow disturbances in small intramyocardial perforating arteries were visible. The coronary flow reserve was reduced to a similar level (around to 2.0) in all three major arteries. In our patient with Anderson-Fabry disease, the coronary vasodilatation was blunted in a diffuse pattern corresponding to the myocardial hypertrophy distribution. In small intramyocardial arteries coronary flow was also disturbed. Accordingly, retrograde systolic flow and accelerated anterograde diastolic flow were documented.

## Background

Anderson-Fabry disease is an X-linked, multisystem, lysosomal storage disease (deficiency of enzyme α-galactosidase A), characterized by the accumulation of glycosphingolipids in various tissues and organs [1,2], including skin, vascular endothelium, heart, kidneys, liver, lungs, pancreas and ganglion cells of the peripheral nervous system. The incidence is 1:117000. However, the rate may be underestimated because a common cardiac manifestation is myocardial hypertrophy that mimics hypertrophic cardiomyopathy. Abnormal storage in the cardiovascular system may also involve cardiac conduction system, valvular apparatus and endothelial cells in coronary vessels [1-3].

To assess coronary flow abnormalities in a patient with Anderson-Fabry disease, we performed transthoracic Doppler echocardiography. Using this method, coronary flow reserve is effectively measurable [4] and all three major coronary arteries are accessible in some patients [5,6]. In a large series of 658 patients [6], coronary flow reserve was contemporarily recorded in left anterior descending (LAD) coronary artery (98% of patients), right coronary artery (RCA) (66% of patients) and circumflex (Cx) coronary artery (43% of patients). Additionally, flow disturbances in small intramyocardial perforating arteries were assessed as blood flow abnormalities at this level of coronary circulation were previously reported in left ventricular (LV) hypertrophy [7].

## Case presentation

This 49-year-old male patient with Anderson-Fabry disease was referred to our hospital. He did not complain of anginal symptoms or dyspnea. As a part of an overall clinical evaluation, transthoracic echocardiography was performed, revealing a diffusely distributed myocardial hypertrophy, i.e. involving both (LV) free walls and the septum (the myocardial thickness at diastole was measured in the short parasternal-axis: the anterior segment of the septum- 19.6 mm; the posterior segment of the septum – 20.8 mm; the LV posterior wall – 20.7 mm; and the anterolateral wall 16.8 mm). Left ventricular systolic function was preserved (LV ejection fraction 68%). A precise assessment of myocardial hypertrophy by magnetic resonance imaging confirmed increased LV mass to 386 grams.

## Methods

**Figure 1:**
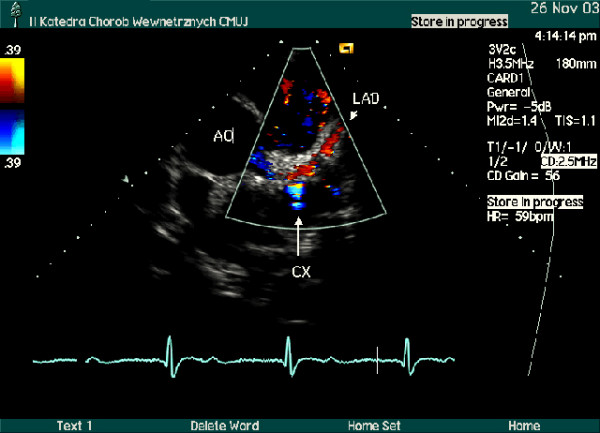
Parasternal short axis view, color Doppler examination at the heart base level. Left main, left anterior descending (LAD) and origin of left circumflex (Cx) coronary arteries are seen. Blood flow within the left main coronary artery and proximal LAD is depicted in red, while in the Cx – in blue.

**Figure 2:**
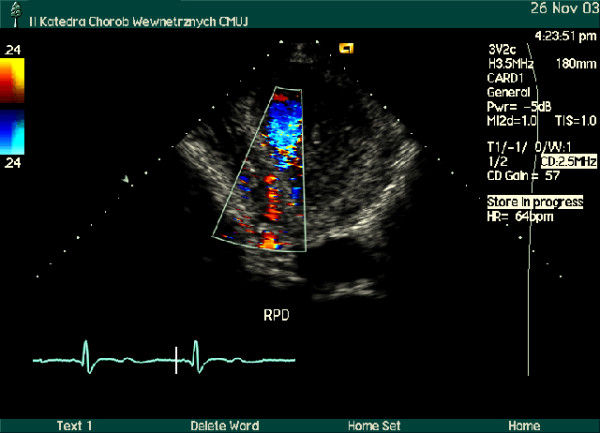
Modified apical four-chamber view, color Doppler examination. Flow detected in long segment of the right posterior descending (RPD) coronary artery is depicted in red.

Using noninvasive, inexpensive and widely accessible method, B-mode and color Doppler transthoracic echocardiography, the segments of three major coronary arteries: LAD, Cx and right posterior descending (RPD) coronary artery or RCA were visualized (Figure 1,2). High quality recordings of flow velocity in all these coronary arteries were obtained using spectral Doppler.

## Results

**Figure 3:**
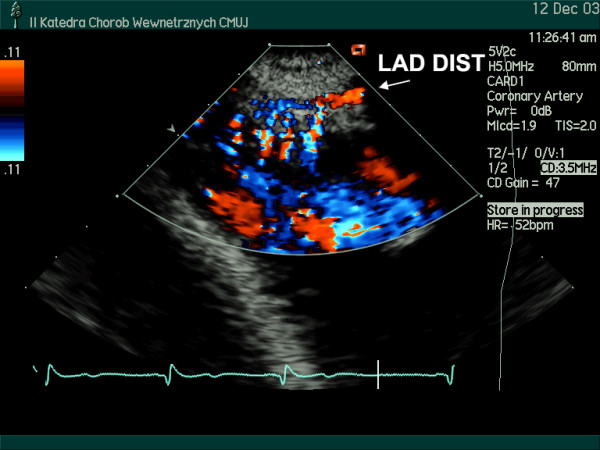
Modified apical 2-chamber view, apical area, color Doppler examination. The LAD and the penetrating intramyocardial arteries (vertical perforators branching of the LAD) are seen.

**Figure 4:**
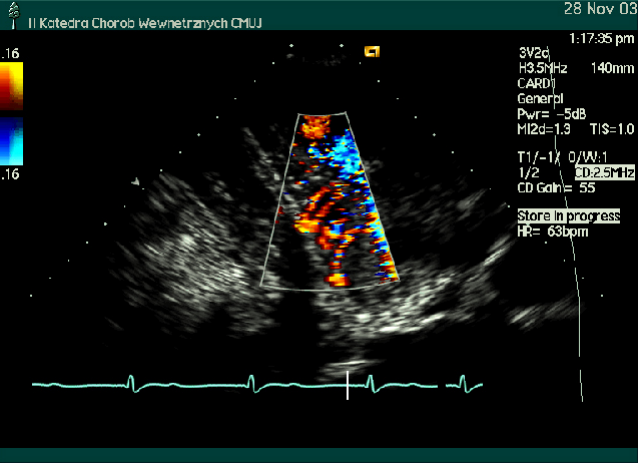
Modified apical 2-chamber view, middle segment of the inferior wall on color Doppler examination. Small (arch-shaped) arteries branching of the right posterior descending (RPD) coronary artery are seen.

**Figure 5:**
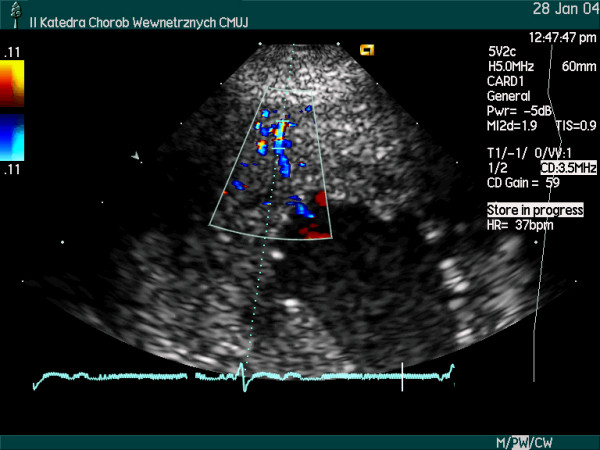
The orientation of Doppler gate within the LAD perforator.

**Figure 6:**
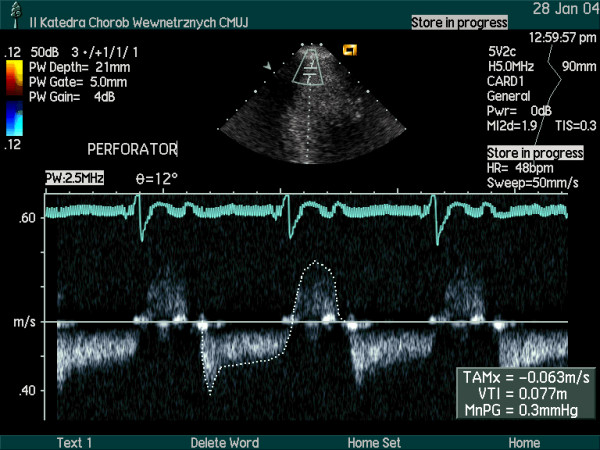
The flow velocity spectrum in the LAD perforator visualized in figure 5. In diastole the flow velocity is negative (towards ventricular chamber) and during systole the retrograde flow (towards epicardium) results in positive value of velocity.

**Figure 7:**
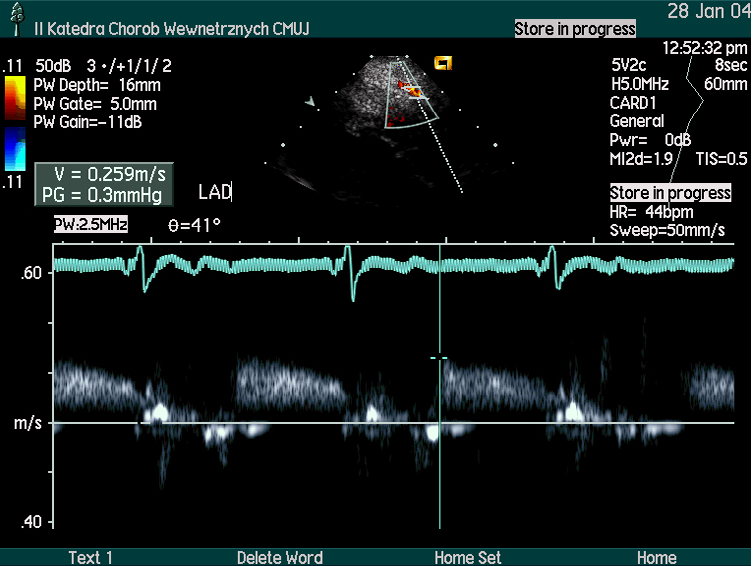
Diastolic flow velocity spectrum in distal portion of LAD.

The coronary flow reserve in response to intravenous adenosine (140 μg/kg/min) was homogeneously reduced to a similar value in the major coronary arteries (LAD – 2.07; Cx- 2.18; RPD/RCA- 1.91). Small, intramyocardial branches of epicardial coronary arteries were visualized [intramyocardial perforators originated from LAD (Figure 3) and branches from RPD (Figure 4)]. Increased resistance to flow (probably due to myocardial hypertrophy and increased extravascular compressive forces) was demonstrated by the detection of flow with high velocity in spectral Doppler (figure 5, 6, 7) and color Doppler (Figures 3, 4, 5) in these penetrating vessels. The peak diastolic flow velocity was higher in the LAD perforator (41 cm/s – figure 6) than in the distal portion of LAD (25 cm/s – figure 7). The systolic flow in the LAD perforator was abnormally retrograde (figure 6).

## Discussion

Prevalence of Anderson-Fabry disease in patients with late-onset hypertrophic cardiomyopathy is about 6.3% in males [8] and 12% in females [9]. In contrast, among males with hypertrophic cardiomyopathy diagnosed at <40 years of age, the rate of appropriate verification of the diagnosis to Anderson-Fabry disease was lower i.e. 1.4% [8]. If properly recognized, Anderson-Fabry disease is treatable by enzyme replacement therapy and both cardiac and non-cardiac abnormalities may be reversed/reduced by substitution of α-galactosidase (especially in the early stage) [10]. Therefore, it is important to consider Anderson-Fabry disease in the differential diagnosis of hypertrophic cardiomyopathy. Anderson-Fabry disease is hardly indistinguishable from hypertrophic cardiomyopathy by echocardiography [9,11,12], however we made an attempt to identify potential differences (mainly quantitative) in table 1. Recently [13], the findings of magnetic resonance imaging have appeared useful in differential diagnosis (table 1). Clinical findings may be more helpful in differential diagnosis and we recommend to evaluate the presence or absence of all non-cardiac manifestations (dermatological, nephrological, neurological, ophthamological) of Anderson-Fabry disease in patients diagnosed as having hypertrophic cardiomyopathy.

**Table 1:** Comparison of echocardiography and MRI findings between Anderson-Fabry and hypertrophic cardiomyopathy.

Echocardiography		
	Anderson-Fabry	HCM

LVH pattern	Concentric>Asymmetric	Asymmetric>Concentric

Resting LVOT gradient	Rare	25–30%

Massive LVH> 30 mm	Rare	10–15%

LV diastolic dysfunction (tissue Doppler, strain rate)		
With LVH	Yes	Yes
Without LVH (genotype +)	Yes	Yes

LV systolic dysfunction	More frequent especially in older males	

Magnetic resonance		

Most common site of late-enhancement (if present)	Infero-lateral segment	Ventricular junction, Multi-focal

LVH- left ventricular hypertrophy; LVOT – left ventricular outflow tract

## Conclusion

In our patient with Anderson-Fabry disease, the coronary vasodilatation was blunted in a diffuse pattern corresponding to the myocardial hypertrophy distribution. In small intramyocardial arteries coronary flow was disturbed. Accordingly, retrograde systolic flow and accelerated anterograde diastolic flow were documented. Transthoracic Doppler echocardiography is now the only method available to evaluate blood flow characteristics in small intramyocardial arteries.

## List of Abbreviations

LAD – left anterior descending coronary artery

RCA – right coronary artery

RPD – right posterior descending coronary artery

Cx – circumflex coronary artery

## Competing Interests

The author(s) declare that they have no competing interests.
